# One‐Year Outcome of Japanese Patients With Atrial Fibrillation: Insights From APHRS‐AF Registry

**DOI:** 10.1002/joa3.70287

**Published:** 2026-02-08

**Authors:** Kenji Yodogawa, Yu‐ki Iwasaki, Yasuo Okumura, Koichi Nagashima, Koichi Inoue, Nobuaki Tanaka, Kengo Kusano, Koji Miyamoto, Masahiko Takagi, Kyoko Soejima, Yuichi Momose, Tomohiro Sakamoto, Hideharu Okamatsu, Toyoaki Murohara, Yasuya Inden, Keiichi Fukuda, Seiji Takatsuki, Yasuki Kihara, Yukiko Nakano, Teiichi Yamane, Michifumi Tokuda, Masayoshi Ajioka, Hiroyuki Osanai, Kazuhiro Satomi, Hiroyuki Tsutsui, Akihiko Shimizu, Satoru Sakagami, Eiichi Watanabe, Nobuhisa Hagiwara, Mitsuharu Kawamura, Naohiko Takahashi, Yoshinori Kobayashi, Hirofumi Tomita, Hiroshi Tada, Kazutaka Aonuma, Yukihiro Koretsune, Takanori Ikeda, Masahiko Goya, Wataru Shimizu

**Affiliations:** ^1^ Department of Cardiovascular Medicine Nippon Medical School Tokyo Japan; ^2^ Division of Cardiology, Department of Medicine Nihon University School of Medicine Tokyo Japan; ^3^ Cardiovascular Center Sakurabashi Watanabe Advanced Healthcare Hospital Osaka Japan; ^4^ Department of Cardiovascular Medicine National Cerebral and Cardiovascular Center Suita Japan; ^5^ Department of Medicine II Kansai Medical University Moriguchi Japan; ^6^ Department of Cardiovascular Medicine Kyorin University Hospital Tokyo Japan; ^7^ Division of Cardiology Saiseikai Kumamoto Hospital Cardiovascular Center Kumamoto Japan; ^8^ Department of Cardiology Nagoya University Graduate School of Medicine Nagoya Japan; ^9^ Department of Cardiology Keio University School of Medicine Tokyo Japan; ^10^ Department of Cardiovascular Medicine Hiroshima University Graduate School of Biomedical and Health Sciences Hiroshima Japan; ^11^ Division of Cardiology, Department of Internal Medicine The Jikei University School of Medicine Tokyo Japan; ^12^ Department of Cardiology Tosei General Hospital Seto Japan; ^13^ Heart Rhythm Center Tokyo Medical University Tokyo Japan; ^14^ Department of Cardiovascular Medicine, Faculty of Medical Sciences Kyushu University Fukuoka Japan; ^15^ Department of Medicine and Clinical Science Yamaguchi University Graduate School of Medicine Yamaguchi Japan; ^16^ National Hospital Organization Kanazawa Medical Center Kanazawa Japan; ^17^ Department of Internal Medicine Fujita Health University Bantane Hospital Nagoya Japan; ^18^ Department of Cardiology Tokyo Women's Medical University Tokyo Japan; ^19^ Division of Cardiology Showa University School of Medicine Tokyo Japan; ^20^ Department of Cardiology and Clinical Examination Oita University Faculty of Medicine Oita Japan; ^21^ Division of Cardiology Tokai University Hachioji‐Hospital Tokyo Japan; ^22^ Department of Cardiology Hirosaki University Graduate School of Medicine Aomori Japan; ^23^ Hirosaki Stroke and Rehabilitation Center Aomori Japan; ^24^ Department of Cardiovascular Medicine, Faculty of Medical Sciences University of Fukui Fukui Japan; ^25^ Division of Cardiology University of Tsukuba Hospital Tsukuba Japan; ^26^ Cardiovascular Division National Hospital Organization Osaka National Hospital Osaka Japan; ^27^ Department of Cardiovascular Medicine Toho University Faculty of Medicine Tokyo Japan; ^28^ Department of Cardiovascular Medicine Tokyo Medical and Dental University Tokyo Japan; ^29^ Department of Cardiovascular Medicine New Tokyo Hospital Matsudo Japan

**Keywords:** Asian, atrial fibrillation, Japan, prospective studies, registries

## Abstract

**Background:**

The Asia‐Pacific Heart Rhythm Society Atrial Fibrillation (APHRS‐AF) Registry is a prospective study in Asian metropolitan cities, which provides important information on the baseline characteristics, therapeutic patterns, and 1‐year clinical outcomes in patients with atrial fibrillation (AF). This report describes data from Japanese patients recruited in this registry.

**Methods and Results:**

A total of 4666 patients with AF were enrolled. Of these, 794 patients were recruited from 28 large cardiovascular centers in Japan between 2015 and 2017. We analyzed 1‐year follow‐up outcome of these patients. Mean age at recruitment was 65.7 years and 69.0% were males. Major comorbidities were hypertension (37.5%), lipid disorder (29.0%), heart failure (15.9%), and diabetes mellitus (15.0%). Mean CHADS2 score, CHA2DS2‐VASc score, and HAS‐BLED score were 1.0, 2.0, and 1.1, respectively. At baseline, use of oral anticoagulants was 81%, including 7% prescribed a vitamin K antagonist (VKA) and 74% a direct oral anticoagulant (DOAC). Majority of the patients (*N* = 459, 57.8%) were planned to undergo catheter ablation. One‐year follow‐up was conducted in 743 patients. One‐year all‐cause mortality was 0.1% (*n* = 1) and the incidence of stroke/thromboembolic events was also 0.1% (*n* = 1). Major bleeding events were observed in 5 patients (0.7%), including 3 intracranial hemorrhages.

**Conclusion:**

In this 1‐year analysis, a high prevalence of oral anticoagulant use was recorded. A low mortality rate and a low incidence of stroke/thromboembolic events were observed in Japanese patients of the APHRS‐AF Registry.

## Graphical abstract

A total of 794 patients recruited from 28 large cardiovascular centers in Japan were prospectively investigated. Patients were relatively young, had low CHADS2 scores, and relatively few comorbidities such as hypertension. Majority of the patients were scheduled for catheter ablation. Short‐term prognosis was found to be extremely favorable.

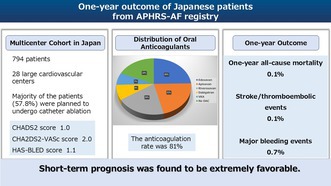

## 1 Introduction

Atrial fibrillation (AF) is the most common arrhythmia in clinical practice and has been reported to be associated with high morbidity and mortality [1].

However, the management of AF has markedly changed after the introduction of direct oral anticoagulant drugs (DOACs) and catheter ablation as a rhythm control strategy [2].

The Asia‐Pacific Heart Rhythm Society Atrial Fibrillation (APHRS‐AF) Registry is a prospective study in Asian metropolitan cities, which provides important information on the baseline characteristics, therapeutic patterns, and 1‐year clinical outcomes in patients with atrial fibrillation (AF) in the modern era of DOACs and catheter ablation [3]. This report describes data from Japanese patients recruited in this registry.

## 2 Methods

This is a multicenter prospective observational study. A total of consecutive 4666 in‐ and out‐ patients who presented with AF to a cardiologist were enrolled in five Asian metropolitan cities (Hong Kong, South Korea, Japan, Singapore, and Taiwan). All patients enrolled had an electrocardiogram (ECG) of AF, including Holter, event monitor or implantable loop recorder within the 12 months prior to enrollment. Of these, 794 patients were recruited from 28 large cardiovascular centers in Japan between 2015 and 2017. After baseline assessment at enrolment, 1‐year follow‐up was performed by the local cardiologist investigator. We evaluated mortality, incidence of stroke/thromboembolism, major bleeding, cardiovascular comorbidities, and hospital readmission within 1 year in these Japanese patients. Major bleeding was defined as bleeding causing a drop of Hemoglobin (Hb) > 2 g/L, requiring blood transfusion and/or (lengthening of) hospital admission. This study was approved by our local Ethical Committee (28–06‐594), and informed consent was obtained from all patients.

### 2.1 Statistical Analysis

Continuous variables were presented as mean value ± standard deviation (SD), and categorical variables were summarized as count and percentage. Kaplan–Meier analysis was used to determine 1‐year all‐cause mortality, stroke/thromboembolic events rate, and major bleeding events rate. All statistical analyses were performed using EZR (Saitama Medical Center, Jichi Medical University, Saitama, Japan) [4].

## 3 Results

### 3.1 Baseline Characteristics

**FIGURE 1:**
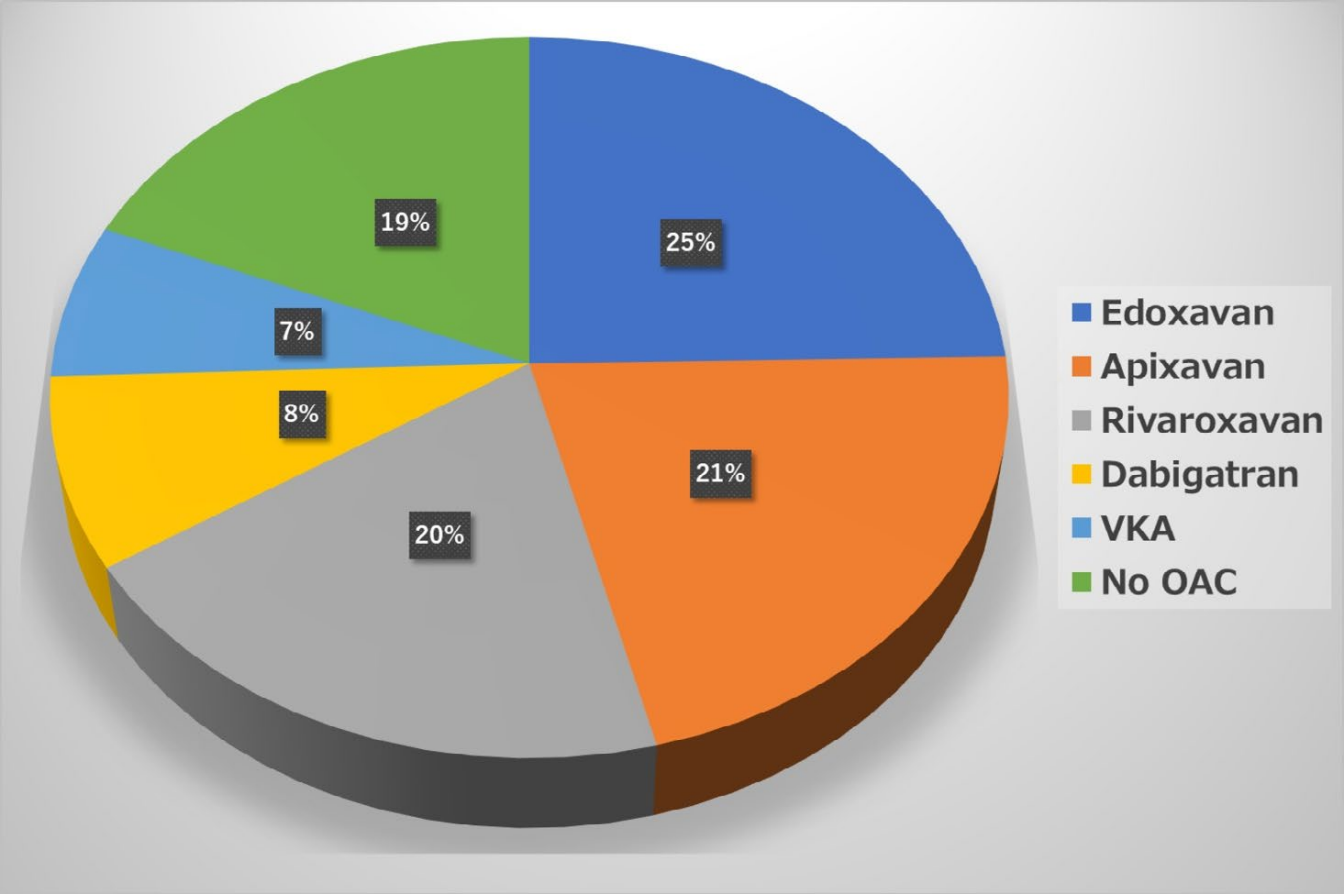
Distribution of oral anticoagulants (OACs) in the study population. VKA, vitamin K antagonist.

Mean age at recruitment was 65.7 years and 69.0% were males. Major comorbidities were hypertension (37.5%), lipid disorder (29.0%), heart failure (16.0%) and diabetes mellitus (15.0%). Mean CHADS2 score, CHA2DS2‐VASc score, and HAS‐BLED score was 1.0, 2.0, and 1.1, respectively. Paroxysmal AF was detected in 464 patients (58.4%), persistent AF in 213 (26.8%), long‐standing AF in 53 (6.7%), permanent AF in 32 (4.0%), and first diagnosed AF in 32 (4.0%) (Table 1). Notably, majority of the patients (*N* = 459, 57.8%) were planned to undergo catheter ablation, and it was performed during the hospitalization following enrollment in 362 (45.6%) patients. At baseline, use of oral anticoagulants was 81%, including 7% prescribed a vitamin K antagonist (VKA) and 74% a direct oral anticoagulant (DOAC) (Figure 1).

**TABLE 1:** Clinical characteristics of the study patients (*n* = 794).

Baseline characteristics	
Age (years)	65.7 ± 10.8
Male	548 (69.0%)
LAD (mm)	40.4 ± 7.4
LVEF (%)	62.3 ± 10.6
HF	127 (16.0%)
HT	298 (37.5%)
DM	120 (15.1%)
Lipid disorder	230 (29.0%)
Stroke/TIA	46 (5.8%)
CAD	47 (5.9%)
CKD	71 (8.9%)
CHADS_2_ score	1.0 ± 1.1
CHA2DS2‐VASc score	2.0 ± 1.6
HAS‐BLED score	1.1 ± 1.0
First diagnosed AF	32 (4.0%)
Paroxysmal AF	464 (58.4%)
Persistent AF	213 (26.8%)
Longstanding persistent AF	53 (6.7%)
Permanent AF	32 (4.0%)
DOAC	591 (74.4%)
VKA	55 (6.9%)
Antiplatelets	53 (6.7%)
AAD	123 (15.5%)
History of catheter ablation for AF	200 (25.2%)
Planned catheter ablation for AF	459 (57.8%)

Abbreviations: AAD, antiarrhythmic drugs; AF, atrial fibrillation; CAD, coronary artery disease; CKD, chronic kidney disease; DM, diabetes mellitis; DOAC, direct oral anticoagulant; HF, heart failure; HT, hypertension; LAD, left atrial daiameter; LVEF, left ventricular ejection fraction; TIA, transient ischemic attack; VKA, vitamin K antagonist.

### 3.2 One‐Year Follow‐Up Outcome

**FIGURE 2:**
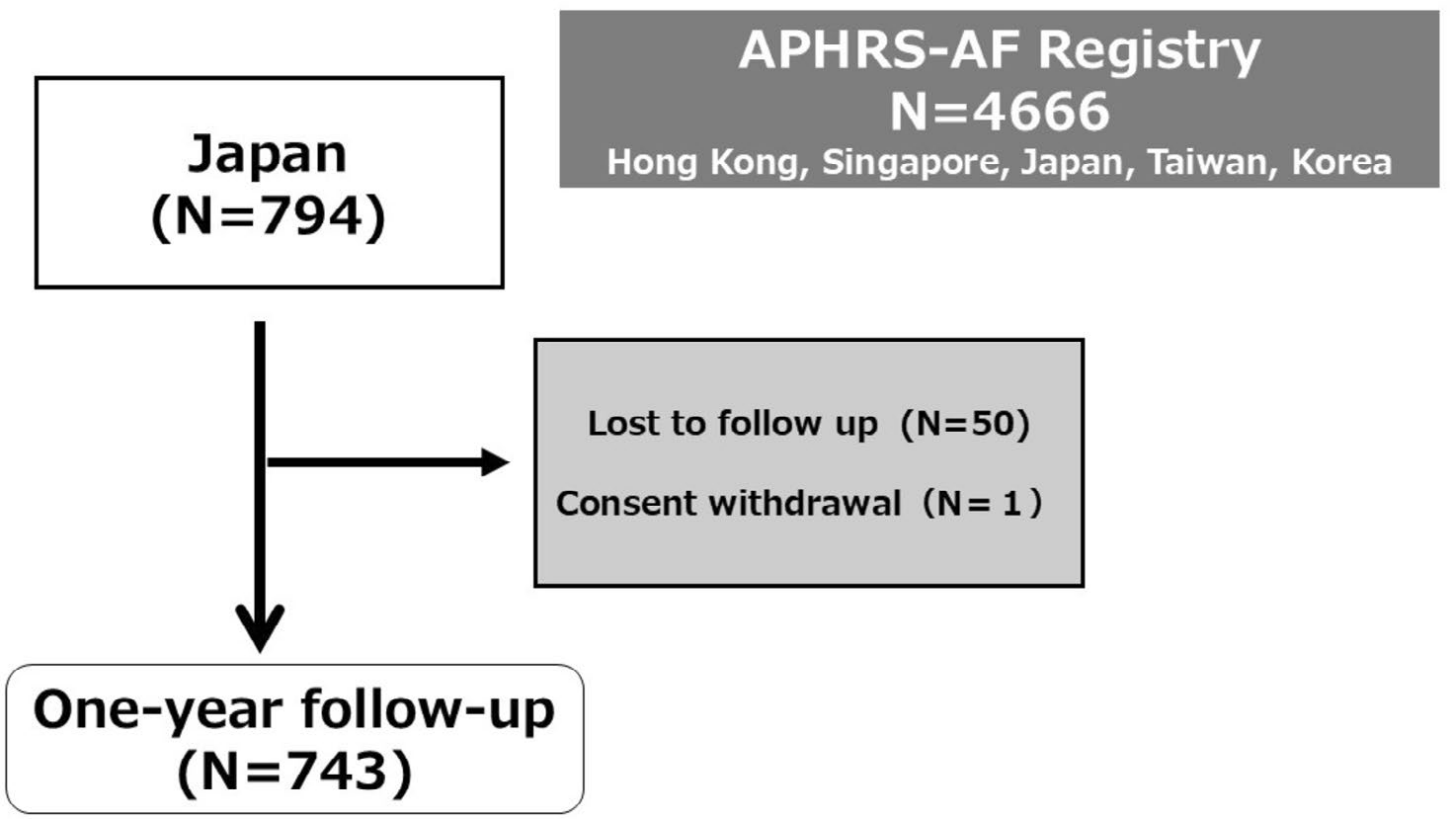
Patient flow of the study.

One‐year follow‐up was conducted in 743 patients (lost to follow up; *N* = 50, consent withdrawal; *N* = 1, Figure 2). One‐year all‐cause mortality was 0.1% (*n* = 1, heart failure), and the incidence of stroke/thromboembolic events was also 0.1% (*n* = 1, pulmonary embolism). The patient with pulmonary embolism was not taking anticoagulation at the time of the event. Major bleeding event rates were 0.7% (*n* = 5, 3 intracranial hemorrhages, 1 lower gastrointestinal bleeding, 1 soft tissue bleeding). One patient with major bleeding was not taking anticoagulation at the time of the event, and the remaining 4 patients were taking DOAC. Clinically relevant nonmajor bleeding was observed in 5 (0.7%) patients. A total of 157 (21.1%) nonfatal hospitalizations were observed. The major causes of hospitalizations were AF, atrial flutter (AFL), or atrial tachycardia (AT) (*n* = 102, 13.7%) (Table 2).

**TABLE 2:** Death and nonfatal hospitalization at 1‐year (*n* = 743).

Clinical events	Cumulative 1 year (/100 person years)
All cause death	1 (0.1)
Cardiac	1
Others	0
Nonfatal hospitalization	157 (21.1)
Stroke/thromboembolic events	1 (0.1)
Intracranial bleeding	3 (0.4)
GI bleeding	1 (0.1)
Bleeding in soft tissue, muscle, skin	1 (0.1)
Clinically relevant nonmajor bleeding	5 (0.7)
AF/AFL/AT	102 (13.7)
Arrhythmias other than AF/AFL/AT	11 (1.5)
HF	4 (0.5)
ACS	2 (0.3)
Other cardiovascular events	6 (0.8)
Noncardiovascular events	21 (2.8)

Abbreviations: ACS, acute coronary syndrome; AF, atrial fibrillation; AFL, atrial flutter; AT, atrial tachycardia; GI, gastrointestinal; HF, heart failure.

## 4 Discussion

The present study demonstrated an extremely low mortality rate in patients with AF recruited in this registry. The incidence of stroke/thromboembolic events was also extremely low. In addition, a low incidence of major bleeding was observed.

### 4.1 Comparison With the Other Japanese Registries

The present registry demonstrated lower mortality (0.1%/year), lower incidence of stroke/thromboembolic events and bleeding events (0.1%, 0.7%, respectively) compared to those in previous Japanese AF registries. The Fushimi AF Registry, the large database of Japanese AF patients, showed 7.9% annual mortality rate. Annual incidence of stroke or systemic embolism and major bleeding were 2.7%, 1.5%, respectively. However, The Fushimi AF Registry is a community‐based cohort, whereas the Japanese APHRS‐AF Registry is cardiovascular center‐based cohort. Discrepancies in the mortality rate, incidence rates are assumed to be derived from differences in patient background. Mean age was higher than that of the present registry (73.6 vs. 65.7 years). Notably, nearly half of the patients were not taking oral anticoagulants (Table 3) [5, 6].

**TABLE 3:** Comparison of Japanese‐APHRS‐AF with other AF registries.

Registry	Japanese APHRS‐AF	APHRS‐AF	Fushimi‐AF	SAKURA‐AF	RYOUMA
Year	2015–2017	2015–2017	2011–2012	2013–2015	2017–2018
Study population	28 large cardiovascular centers in Japan	52 centers in 5 countries with a broad mix oftertiary and general hospitals	79 institutions including 67 primary‐care clinics in Japan	2 cardiovascular centers, 13 affiliated or community hospitals, 48 private clinics	Patients with a planned first catheter ablation for AF in 62 institutions
Number of patients	794	4664	3731	3266	3072
Age (years)	65.7 ± 10.8	68.5 ± 11.8	73.6 ± 11.0	72.0 ± 9.4	68.0 [60.0–74.0]
Male (%)	69.0	65.5	59.3	74.3	71.1
Paroxysmal AF (%)	58.4	42.4	49.0	37.0	64.2
Comorbidities					
HT (%)	37.5	60.9	62.1	71.3	61.2
Lipid disorder (%)	29.0	37.8	43.2	38.7	NA
HF (%)	16.0	20.8	26.9	22.1	NA
DM (%)	15.1	24.2	22.9	22.8	17.3
CKD (%)	8.9	7.6	35.2	24.6	9.6
Prior stroke/TIA (%)	5.8	9.6	18.3	11.2	NA
CHADS2 score	1.0 ± 1.1	NA	2.0 ± 1.3	1.8 ± 1.2	1.0 [0.0–2.0]
CHA2DS2‐VASc score	2.0 ± 1.6	2.2 ± 1.6 (Males) 3.6 ± 1.6 (Females)	3.4 ± 1.7	2.7 ± 1.4	2.0 [1.0–3.0]
HAS‐BLED score	1.1 ± 1.0	1.3 ± 1.0 (Males) 1.5 ± 1.0 (Females)	1.6 ± 0.9	1.4 ± 0.9	2.0 [1.0–3.0]
Oral anticoagulants					
DOACs (%)	74.4	79.8	7.2	51.7	92.6
VKA (%)	6.9	17.8	46.3	48.3	5.1
Event rate per 100 patient‐years					
Death	0.1	2.7	7.9	2.1	0.5
Stroke/thromboembolic events	0.1	0.7	2.3	1.4	0.3
Major bleeding	0.7	1.1	1.8	1.3	1.2

*Note:* Data are shown as mean ± SD, or median [IQR, Q1–Q3] or *n* (%).

Abbreviations: AF, atrial fibrillation; CKD, chronic kidney disease; DM, diabetes mellitus; DOAC, direct oral anticoagulant; HF, heart failure; HT, hypertension; TIA, transient ischemic attack; VKA, vitamin K antagonist.

SAKURA AF Registry is a recent prospective observational registry in patients with documented AF being treated with anticoagulants. In total, 3266 patients were enrolled at 2 university hospitals, 13 affiliated or community hospitals, and 48 private clinics between September 2013 and December 2015. In this registry, annual mortality rate was 0.64%, and annual incidence of stroke or systemic embolism and major bleeding were 1.17%, 1.21%, respectively [7, 8]. These incidences were higher than those in our registry, and this may also be due to differences in patient background. Mean age was higher than that of the present registry (72.0 vs. 65.7 years), and a higher proportion of patients had hypertension compared with our study (71.3% vs. 37.5%) (Table 3).

On the other hand, in Japanese patient cohorts like those in the APHRS‐AF registry—who were relatively young, had low CHADS2 scores, and few comorbidities such as hypertension and were scheduled for catheter ablation at large cardiovascular centers—short‐term prognosis was found to be extremely favorable.

RYOUMA registry is a prospective multicenter observational study of Japanese patients who underwent catheter ablation for AF in 2017–2018. The incidence of thromboembolic and major bleeding events for 1 year was 0.3% and 1.2%, which were nearly consistent with those in the present study [9].

### 4.2 Comparison With the APHRS AF Registry Including 5 Countries

In comparison with the whole APHRS‐AF Registry [3], we observed an overall lower mortality rate (0.1% vs. 2.7%). The reasons remain unclear because non cardiovascular rather than cardiovascular events were the most common causes of mortality in the whole APHRS‐AF Registry. However, it might be partially attributed to the younger population (65.7 ± 10.8 vs. 68.5 ± 11.8 years) in Japanese APHRS‐AF Registry. Moreover, Japanese APHRS‐AF Registry showed lower proportion of heart failure (16.0% vs. 21.2%) and coronary artery disease (5.9% vs. 19.5%) compared to those in whole APHRS‐AF Registry [10], which also might attribute to the lower mortality. Furthermore, Bucci et al. recently reported that diabetes mellitus (DM) was independently associated with a higher risk of all‐cause death, cardiovascular death, and major bleeding in the whole APHRS‐AF Registry. Our registry showed lower proportion of DM compared to those in the whole APHRS‐AF Registry (15.1% vs. 24.6%), which might affect the mortality [11]. The Japanese APHRS‐AF Registry demonstrated lower incidence of stroke/thromboembolic events and bleeding events compared to those in whole APHRS‐AF Registry (0.1% vs. 0.7%, 0.7% vs. 1.7%, respectively). While the reasons remain unclear, it is possibly influenced by the lower CHA2DS2‐VAScscore and HAS‐BLED score in the Japanese APHRS‐AF Registry compared to those in whole APHRS‐AF Registry [11].

Recently, Chao TF et al. described that significant differences of clinical features were noted between countries in APHRS‐AF Registry. The mean age of AF patients in Hong Kong was 73.59 years compared to 65.80 years in Japan. In addition, the comorbidities were diverse, with the highest CHA2DS2‐VASc scores reported from Hong Kong (mean 3.5) and lowest in Japan (mean 2.0). The HAS‐BLED score showed significant variation, with the highest mean in Hong Kong (1.8) and the lowest in Japan (1.1) [12]. These differences in profiles of patients enrolled by cardiologists at medical centers may contribute to the low mortality and low incidence of stroke/thromboembolic events in Japan.

## 5 Study Limitations

There are limitations in this study. First, this cohort was followed by electrophysiologists with a high proportion of patients undergoing catheter ablation (57.8%), which limits generalizability to the broader Japanese AF population. This might partly account for the low mortality and low incidence of stroke/thromboembolic events and bleeding events. Second, about 6% of our patients were lost to follow‐up. However, this value is lower than the 12% observed in the whole APHRS‐AF Registry [3]. Third, similar to the whole APHRS‐AF Registry, we did not have data on International Normalized Ratio (INR) control in our patients treated with VKA [3]. Therefore, it remains unclear whether the INR control in patients treated with VKA affected their outcomes. However, we did not observe any significant thromboembolic or bleeding events among those treated with VKA.

## 6 Conclusions

In this registry from large cardiovascular centers, the majority of the patients were planned to undergo catheter ablation, and a high prevalence of oral anticoagulant use was recorded. A low mortality rate and low incidence of stroke/thromboembolic events were observed in Japanese patients of the APHRS‐AF Registry. However, the findings are limited by focusing on large cardiovascular centers, which may not fully reflect the broader Japanese AF population. Future studies with extended follow‐up durations including both private and public sector patients are warranted to validate these insights into AF management in Japan.

## Funding

This study was an independent research grant by Pfizer and Bristol Myers Squibb (BMS) to Asia‐Pacific Heart Rhythm Society.

## Ethics Statement

This study was approved by the institutional ethics committee of Nippon Medical School (Approval No. 28‐06‐594). Informed consent was taken from patients.

## Conflicts of Interest

Yasuo Okumura has received research funding from Medtronic Japan Co. Ltd., MicroPort CRM Japan, and Bayer Healthcare; and has accepted remuneration from AstraZeneca K.K. and Johnson & Johnson K.K. He is affiliated with endowed departments supported by Abbott Japan LLC, Boston Scientific Japan K.K., Medtronic Japan Co. Ltd., Japan Lifeline Co. Ltd., and Biotronik Japan. Koichi Nagashima has received speaker honoraria from Johnson & Johnson and Medtronic Japan. Koichi Inoue has received remuneration from DAIICHI SANKYO COMPANY Ltd., Medtronic, Boston Scientific, and Bristol Myers Squibb. K. Nobuaki Tanaka has received Honorarium from Bayer, Johnson & Johnson K.K., Nippon Boehringer Ingelheim Co. Ltd., AstraZeneca K.K. and Medtronic Japan Co. Ltd. Kengo Kusano has received speaker honoraria from DAIICHI SANKYO COMPANY Ltd., and Medtronic Japan, and research grants from Medtronic Japan, Abott Japan, Boston Scientific Japan, Biotronik Japan, GE Precision Healthcare LLC, Johnson & Johnson K.K. and JSR. Koji Miyamoto has received funding/grants from Bristol‐Myers Squibb, Medtronic, Biosense Webster, Abbott, and Boston and honoraria/speakers' bureaus from Medtronic, Biosense Webster, Abbott and Boston outside the submitted work, and is affiliated with a department endowed by Medtronic outside the submitted work. Masahiko Takagi has received speaker honoraria from DAIICHI SANKYO COMPANY Ltd., Biotronik Japan, Japan Lifeline Co. Ltd., and Medtronic Japan, and research grants from Japan Lifeline Co. Ltd. and Abott Japan. Kyoko Soejima has received speaker honoraria from Abott Japan and Medtronic Japan. Toyoaki Murohara received lecture fee from Boehringer Ingelheim Japan, AstraZeneca, Viatris, Kowa, Novartis Pharma. He is a member of Circulation Journal's Editorial Board. Seiji Takatsuki belongs to Advanced Cardiac Arrhythmia Therapeutics Endowed Research Course, which is supported by Medtronic Japan, Japan Lifeline, Boston Scientific Japan, Abbott Japan and Biotronik Japan. He has received lecture fees from Medtronic Japan, Japan Lifeline, DAIICHI SANKYO COMPANY Ltd., Boston Scientific Japan, Abbott Japan. Keiichi Fukuda holds shares of Heartseed Inc., and has received remuneration from the company. Yukiko Nakano is a member of Circulation Journal's Editorial Board. Teiichi Yamane has received speaker honoraria from Medtronic Japan and BEG company, and research grants from Japan Lifeline. Michifumi Tokuda has received honoraria from Medtronic Japan, and research grants from Japan Lifeline. Hiroyuki Osanai has received honoraria from DAIICHI SANKYO COMPANY Ltd. Kazuhiro Satomi has received lecture fee from DAIICHI SANKYO COMPANY Ltd. Eiichi Watanabe has received consulting fee from Fukuda Denshi. Naohiko Takahashi has received remuneration from DAIICHI SANKYO COMPANY Ltd., AstraZeneca, Bayer, Boehringer Ingelheim Japan, TOA EIYO LTD., Otsuka Pharmaceutical Co. Ltd., Novartis Pharma, Bristol‐Myers Squibb, Pfizer Inc., and Medtronic. He is a member of Circulation Journal's Editorial Board. Hirofumi Tomita is a concurrent professor of an endowment department supported by Medtronic Japan Co. Ltd. and Boston Scientific Japan Co. Ltd. He has received a speakers' bureau/honorarium from Bayer, Daiichi‐Sankyo, and Bristol‐Myers Squibb. Hiroshi Tada received honoraria for lectures or speakers bureaus from DAIICHI SANKYO COMPANY Ltd.; Novartis Pharma K.K.; Medtronic Japan Co. Ltd., BIOTRONIK Japan Inc., Bristol Myers Squibb, Boston Scientific Japan K.K. He has received grants (Investigator‐initiated study unrelated to the manuscript topic) from Abbott Medical Japan LLC, DAIICHI SANKYO COMPANY Ltd., Nippon Boehringer Ingelheim Co. Ltd.; Otsuka Pharmaceutical Co. Ltd.; Eli Lilly Japan K.K.; Marubun Tsusyo K.K. He is a member of Circulation Journal's Editorial Board. Yukihiro Koretsune has received speaker honoraria from DAIICHI SANKYO COMPANY Ltd. Masahiko Goya has received lecture fee from Johnson & Johnson, Japan Lifeline and Abbott Medical Japan. Wataru Shimizu has received remuneration from Daiichi Sankyo, Boehringer‐Ingelheim, Pfizer, Bristol‐Myers Squibb, Janssen, Johnson & Johnson, Boston Scientific, Japan Life Line, Abbott Japan, and Medtronic Japan. He is a member of Circulation Journal's Editorial Board.

## Data Availability

The deidentified participant data will not be shared.
